# Biotic Transformation of Abiotically Stable Nanoscale UiO-66 Metal–Organic Framework by *Daphnia magna* Results in Chronic Reproductive Toxicity

**DOI:** 10.1021/acsnano.5c16532

**Published:** 2025-12-03

**Authors:** Swaroop Chakraborty, Pankti Dhumal, Iuliia Mikulska, Sang Pham, Laura-Jayne Ellis (Bradford), Dhruv Menon, Superb K. Misra, Iseult Lynch

**Affiliations:** † School of Geography, Earth & Environmental Sciences, 1724 University of Birmingham, Edgbaston B15 2TT, U.K.; ‡ Centre for Environmental Research and Justice (CERJ), University of Birmingham, Edgbaston B15 2TT, U.K.; § Diamond Light Source, Harwell Science and Innovation Campus, Didcot OX11 0DE, U.K.; ∥ Facility of Electron Microscopy, University of Birmingham, Edgbaston B15 2TT, U.K.; ⊥ Department of Chemical Engineering & Biotechnology, 2152 University of Cambridge, Cambridge CB3 0AS, U.K.; # Materials Engineering, 242275 Indian Institute of Technology, Gandhinagar 382355, India

**Keywords:** metal−organic frameworks (MOFs), UiO-66, *Daphnia magna*, biotic transformation, chronic reproductive toxicity, environmental risk assessment, synchrotron X-ray absorption spectroscopy

## Abstract

Metal–organic frameworks (MOFs) are entering water technologies on the premise that abiotic stability predicts ecological safety. We overturn this assumption by showing that UiO-66 – often regarded as chemically and structurally robust – remains intact after 7-day aging in natural borehole water yet undergoes rapid *in vivo* transformation in *Daphnia magna*. Synchrotron Microfocus X-ray absorption spectroscopy (XAS) revealed collapse of the ordered Zr–carboxylate coordination into disordered Zr–O environments within the gut; Extended X-ray Absorption Fine Structure (EXAFS) showed loss of second-shell features, and Transmission Electron Microscopy (TEM) confirmed loss of crystallinity with nanoscale aggregates appearing within 24 h of ingestion. Although acute immobilization was limited (48 h EC_50_ ≈ 26.5 μg mL^–1^), a sublethal, environmentally relevant exposure (10 μg mL^–1^) caused pronounced chronic effects: brood initiation was delayed by 3–5 days and cumulative reproduction decreased by ∼74% without mortality. We attribute these outcomes to gut-level transformation and associated energetic/physiological burdens, not captured by standard acute tests. These results show that abiotic stability does not necessarily imply biological inertness and highlight the need to integrate *in vivo* transformation pathways with chronic end points in environmental risk assessment for water-sector materials. This perspective provides a mechanistic basis to inform Safe-and-Sustainable-by-Design (SSbD) MOFs before widespread deployment in water treatment.

## Graphical abstract

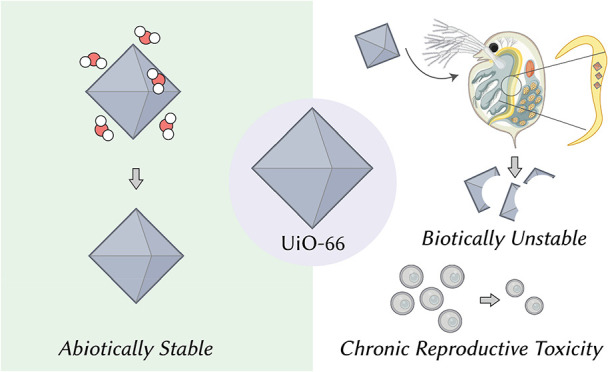

## 1 Introduction

Metal–organic frameworks (MOFs) are an emerging class of porous crystalline materials with diverse applications ranging from gas storage to drug delivery and water remediation.^1–3^ Among them, **UiO-66** – a zirconium (Zr)-based MOF with terephthalate linkers – has gained prominence for environmental uses (e.g., pollutant adsorption) due to its exceptional chemical and thermal stability.^4^ The strong Zr–O coordination bonds endow **UiO-66** with high water stability and low solubility, leading to a common assumption that it is relatively inert or biocompatible. Indeed, prior studies have noted that Zr-containing MOFs tend to exhibit lower acute toxicity compared to MOFs of more labile metals: for example, nanosized **UiO-66** caused no acute lethality in zebrafish embryos (though it did induce yolk sac edema), whereas less stable Zn-based MOFs (ZIF-7/ZIF-8) significantly reduced embryo survival.^5^ Likewise, inert metal oxides like zirconium oxide (ZrO_2_) nanoparticles show 48 h *D. magna* EC_50_ values above 400 mg/L, indicating minimal acute toxicity; yet even these “benign” nanoparticles can impair crustacean reproduction at chronic exposure, with 50% decrease in offspring at ∼ 96 mg/L and a no observed effect concentration (NOEC) as low as 0.78 mg/L.^6^ These findings suggest that robust materials like **UiO-66** may have subtle, longer-term biological effects that are not apparent from short-term assays. Recent research on MOFs in aquatic systems highlights these nuanced effects. Li et al. found that **UiO-66-NH**_**2**_ (an amine-functionalized analogue of UiO-66) caused little direct algal mortality or growth inhibition up to 20 mg/L, yet it stimulated cyanobacteria to release harmful metabolites at concentrations as low as 0.02 mg/L.^7^ In other words, a MOF can elicit stress responses (e.g., toxin release or metabolic disruption) in organisms even when classical toxicity metrics (survival, growth) show no strong effect. Such subtle biochemical impacts underscore why comprehensive chronic studies are needed. For **UiO-66** in particular, which is likely to be encountered by aquatic biota due to its proposed use in water treatment, pollutant adsorption,^8^ and catalysis^9^ in aqueous environments, it is critical to evaluate whether prolonged exposure could impair vital functions like reproduction, even if short-term lethality is minimal.

*D. magna*, a keystone zooplankton species, is widely used as an indicator organism in aquatic toxicology. Standardized tests (OECD 202 for acute immobilization and OECD 211 for chronic reproduction) enable evaluation of both acute and chronic/reproductive impacts of contaminants on *D. magna* populations.^10^ Given the increasing interest in **UiO-66**’s use in environmental applications (such as water treatment, pollutant adsorption, catalysis) and its potential release into aquatic systems,^11^ it is essential to understand how this chemically robust MOF interacts with biological systems.

In this study, we hypothesize that despite its structural stability, **UiO-66** may undergo biotic transformation when exposed to natural waters and aquatic organisms. Here, we assess the behavior of **UiO-66** in natural borehole water and within the D. magna gut, employing microfocus X-ray absorption spectroscopy (XAS) to probe whether the organism can alter **UiO-66**’s local coordination environment. To evaluate potential ecological consequences of such transformation, we conducted standardized ecotoxicological tests: acute immobilization assays (OECD 202)^12^ to establish short-term toxicity thresholds and a 21-day chronic life-cycle test (OECD 211)^13^ at a sublethal concentration to assess effects on survival, maturation and reproduction. Previous studies on MOF toxicity in both ecotoxicological and biological contexts have relied on static end points (e.g., viability, histopathological changes) after exposure or administration,^14,15^ without directly monitoring the structural integrity or chemical transformations of the MOF within the organism. In our approach, we track the structural and chemical evolution of the MOF under quasi-realistic exposure conditions using advanced characterization techniques such as microfocus XAS and X-ray fluorescence (XRF) microscopy,^16^ allowing us to link biotransformation processes to the observed biological effects. This provides several comparative advantages over conventional toxicity assays: (**1**) it offers deeper mechanistic insight into how MOF transformation underpins toxicity, rather than only reporting phenomenological outcomes; (**2**) it allows us to distinguish the contributions of intact particles vs degradation products; and (**3**) it suggests an approach that could be adapted to other nanoporous materials to support mechanism-informed safety assessment. By combining structural characterization with organism-level assays, this study aims to provide new insights into how a chemically and structurally stable MOF may transform under biological influence and what implications this could have for environmental risk assessment.

## 2 Results and Discussion

### 2.1 Physicochemical Characterization, Stability And Abiotic Transformation of **UiO-66**

Transmission Electron Microscopy (TEM) and Scanning Electron Microscopy (SEM) data confirm that the synthesized **UiO-66** consists of uniform faceted cubic morphology (Figure S1a,d). The particles show sharp homogeneous edges, indicative of a well-crystallized framework. Powder X-ray diffraction (PXRD) of the pristine material matches the reference **UiO-66** pattern with no extra reflections (Figure S1c, **lower**), confirming phase purity. Attenuated Total Reflectance – Fourier Transform Infrared (ATR-FTIR) spectroscopy exhibits the expected linker vibrations ν­(C=O) of carboxylate, aromatic C=C and ν­(C–O) – together with Zr–O­(H) modes (Figure S1e, **lower**), again consistent with a defect-lean, crystalline **UiO-66**.^17^

After 7 days in natural bore hole water (BHW) the morphology is retained, albeit with mild agglomeration and subtle surface roughening (Figure S1b). The PXRD peaks characteristic of **UiO-66** persist (Figure S1c, **upper**); the slight attenuation/increased background is consistent with surface hydration rather than a phase change. In the ATR-FTIR spectrum (Figure S1e, **upper**) the linker bands remain, while an enhanced – OH/H_2_O envelope and modest intensity near ∼1360 to 1400 cm^–1^ indicate adsorption of water/hydroxyls and possible carbonate uptake from BHW. High-resolution X-ray photoelectron spectroscopy (XPS) spectra corroborate these surface-level changes (Figure S2a–f). The Zr 3d doublet remains at ∼182 to 183 eV (3d_5/2_) and ∼184.9 to 185.1 eV (3d_3/2_) for both pristine and BHW-aged samples (Figure S2a, d), consistent with Zr (IV) and excluding Zr reduction.^18^ The O 1s region deconvolves into node μ_3_-O/μ_3_–OH and carboxylate O at ∼531 to 533 eV, plus adsorbed H_2_O/–OH at ∼533 to 534 eV; the latter increases after BHW aging (Figure S2b,e), evidencing greater hydration/hydroxylation. In C 1s, the O–C=O component at ∼288.6 to 289.0 eV grows and a shoulder near ∼289 eV becomes more evident (Figure S2c,f), consistent with carbonate/bicarbonate adsorption and/or surface carboxylate enrichment. Together, XPS data indicates that BHW exposure primarily alters the outermost surface, not the Zr oxidation state or the underlying framework.^19,20^

These conclusions align with the Zr K-edge XAS comparisons in Figure 1e,f. The X-ray adsorption near-edge structure (XANES) spectrum of BHW-aged **UiO-66** closely overlaps the pristine spectrum (Figure 1e), indicating that the average Zr local environment is largely preserved under abiotic aging. In the extended fine-Auger structure (EXAFS) spectrum (Figure 1f), the BHW sample shows only modest damping of the first-shell Zr–O and second-shell Zr–Zr features relative to the pristine spectrum, again consistent with minor surface hydration/carbonation rather than node breakdown. Across complementary techniques, **UiO-66** shows crystallographic stability in BHW with surface-level hydration and carbonate adsorption (XPS/ATR-FTIR) and only minor EXAFS damping.

**Figure 1:**
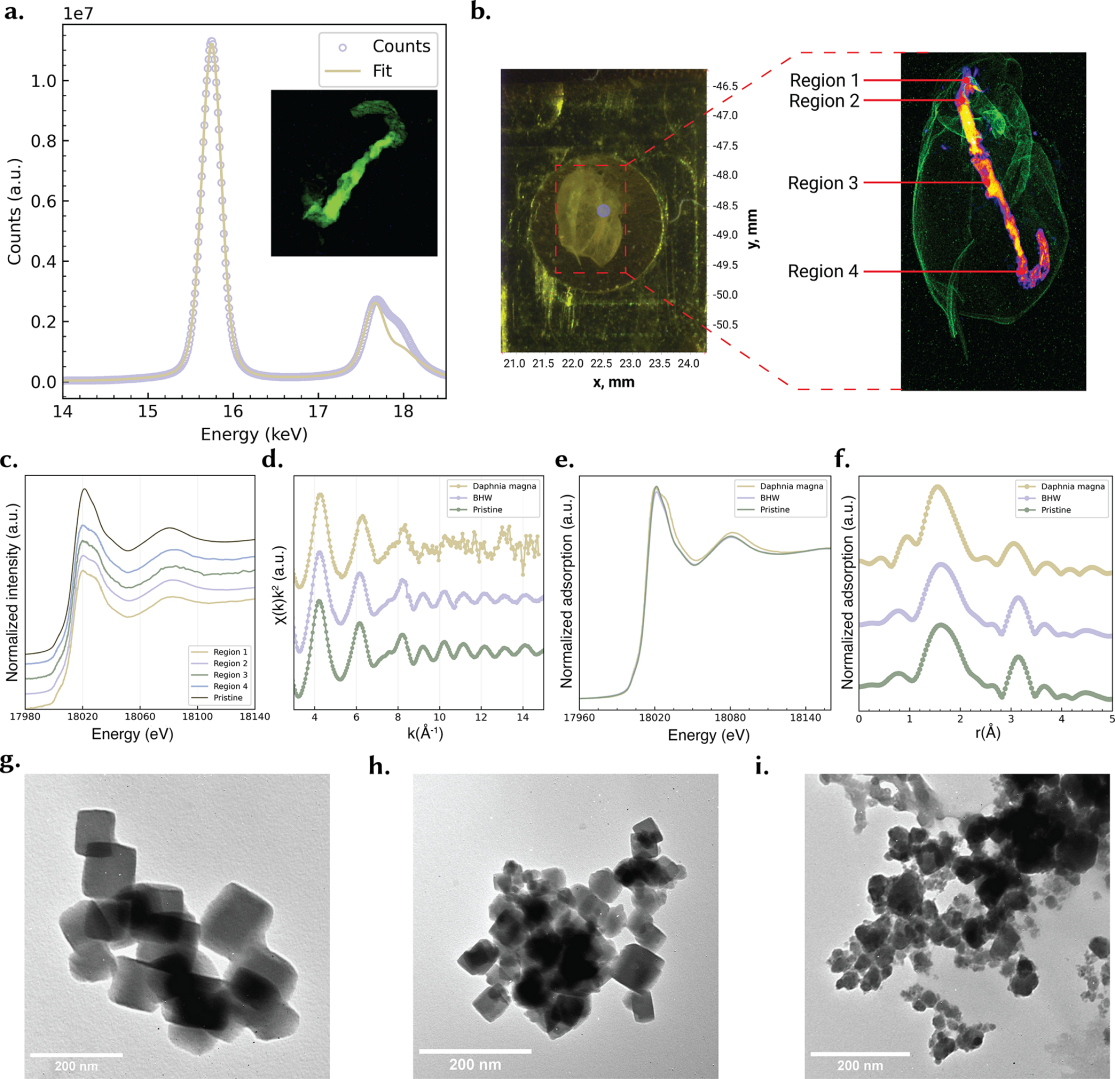
Biotic Transformation and localization of UiO-66 in *D. magna.* (a) XRF spectrum showing Zr Kα emission from **UiO-66** with best-fit curve (inset - XRF map of Zr distribution within *D. magna*). (b) Micro-XRF mapping highlighting Zr distribution across the organism, with four representative regions selected for XAS analysis. **Zr** is rendered with the **Fire** LUT (warm colors = higher counts) and is strongly confined to the digestive tract (gut lumen/epithelium), consistent with ingestion and gut-level processing of the MOF. **Ca** is rendered with the **Green** LUT and delineates the calcified carapace and appendages, providing an anatomical reference (see Figure S7) (c) Normalized XANES spectra comparing pristine **UiO-66** spectrum with spectra obtained from multiple regions of exposed daphnid. (d) EXAFS χ­(k) oscillations showing structural changes between pristine **UiO-66**, **UiO-66** exposed in BHW, and **UiO-66** internalized in *D. magna* gut. (e) Normalized XANES spectra and (f) Fourier-transformed EXAFS spectra confirming partial loss of crystallinity and changes in local coordination environment of Zr upon environmental and biological exposure. (g–i) TEM micrographs showing pristine **UiO-66** crystals (g), agglomerates after 7d exposure in BHW (h), and fragmented/disordered particles after interaction with *D. magna* gut, i.e., following depuration of the daphnid gut contents into clean medium (i).

**UiO-66** exhibited moderate, condition-dependent suspension stability (Figure S8) across the OECD TG-318 screening matrix (pH 4/7/9; Ca­(NO_3_)_2_ = 0/1/10 mM; room temperature (RT) and 37 °C). Stability was quantified as the fraction remaining in the top layer at 6 h after the ∼1 μm centrifugation cutoff, normalized to the measured 0 h value, with Zr determined by ICP-MS. At pH 9, stability increased with ionic strength and temperature, from ∼41 ± 4% (0 mM, RT) to ∼76 ± 8% (10 mM, RT), and from ∼55 ± 6% (0 mM, 37 °C) to ∼88 ± 6% (10 mM, 37 °C) (Figure S8c). At pH 7, a minimum occurred at 1 mM Ca^2+^ (RT ∼ 55 ± 5%; 37 °C ∼ 50 ± 6%), with higher stability at 10 mM (RT ∼ 70 ± 7%; 37 °C ∼ 76 ± 6%) and a strong temperature effect at 0 mM (RT ∼ 55 ± 6% vs 37 °C ∼ 79 ± 4%) (Figure S8b). At pH 4, stability was generally higher at 37 °C (∼69 to 75%) than RT (∼58 to 65%), with a slight dip at 1 mM for both temperatures (Figure S8a). No condition reached ≥90% at 6 h; therefore, **UiO-66** is classified as condition-dependent rather than uniformly “high” by TG-318. Three trends emerge. *(i) Temperature:* Raising the temperature to 37 °C improved retention at all pH/ionic strength combinations. Mechanistically, lower viscosity and faster Brownian motion can help maintain smaller agglomerates in the supernatant during the 6 h window, while temperature-dependent surface charge and hydration also modulate interparticle forces. *(ii) pH:* Alkaline conditions favor stability; at pH 9, stronger deprotonation of the surface carboxylate/μ–OH groups enhances electrostatic repulsion, particularly evident at 0–10 mM Ca^2+^. *(iii) Ionic strength:* The Ca^2+^ dependence is nonmonotonic at pH 4–7, with a minimum near 1 mM that is consistent with partial double-layer compression and Ca^2+^ bridging between surface sites, promoting agglomeration. At 10 mM, retention rises again – most pronounced at pH 7–9 suggesting a shift in the balance of forces (compressed double layers, altered ion correlations, and reduced secondary-minimum trapping) that keeps more material in the upper layer after the ∼1 μm cutoff. We emphasize that these inferences concern hydrodynamic behavior over 6 h and do not imply changes to the underlying framework chemistry.

Therefore, in our study, *“abiotically stable”* refers specifically to the chemical and structural robustness of **UiO-66** in the natural water (i.e., retention of crystallinity and Zr–carboxylate coordination during abiotic aging). The OECD TG-318 measurements presented here address a different property – hydrodynamic suspension stability and show that **UiO-66** exhibits condition-dependent dispersion behavior despite its unusual chemical robustness among MOFs. From an environmental testing standpoint, **UiO-66** would not be described as “suspension stable” in the strict OECD TG-318 sense (≥90% under all screening conditions). However, practically high retention is attainable in alkaline, higher-temperature, higher-ionic-strength scenarios (e.g., pH 9, 10 mM Ca^2+^, 37 °C ≈ 88%), whereas more neutral/acidic and mildly saline conditions (especially ∼ 1 mM Ca^2+^) favor agglomeration and loss from the top layer. These insights help interpret downstream fate and toxicity experiments by identifying media in which **UiO-66** remains better dispersed versus conditions that promote settling/agglomeration.

### 2.2 Biotic Transformation of **UiO-66** MOFs in *D magna gut*

Daphnids were acutely exposed to 100 μg mL^–1^ preaged **UiO-66** suspended in BHW for 24 h, after which intact organisms were analyzed using synchrotron-based XAS techniques. XRF elemental mapping provided the first line of evidence for *in vivo* localization of these MOFs. As shown in Figure 1a, Zr signals were highly enriched (Figure S7) in the digestive tract, particularly the gut lumen and surrounding epithelial tissue (Figure 1b). This distribution pattern suggests that while **UiO-66** is efficiently ingested, it is not widely translocated to peripheral organs. The confinement of Zr within the gut indicates that any subsequent transformation processes are spatially restricted to this compartment, where enzymatic activity, organic acids, and phosphate-rich digestion fluids provide a highly reactive microenvironment. This observation is consistent with prior nanoparticle exposure studies in *D. magna*, where metals frequently accumulated in the midgut or hepatic ceca, resulting in tissue stress and damage.^21^

Importantly, XANES data confirmed that the **UiO-66** ingested by *D. magna* did not remain in its pristine structural state. The Zr K-edge spectra obtained from different gut regions (Regions 1–4, Figure 1c) were virtually indistinguishable, implying that wherever Zr accumulated, it existed in a chemically uniform form. This strongly suggests that **UiO-66** undergoes a systematic and reproducible transformation inside the digestive tract. Compared to the reference spectra, the daphnid-exposed sample exhibited a broadened and less intense white-line feature (Figure 1d). In pristine **UiO-66** and even in BHW-aged samples, the white line is sharp and intense, characteristic of well-ordered Zr–carboxylate coordination within the Zr_6_O_4_ clusters. The suppressed and broadened white line in the daphnid spectrum reflects a breakdown of this order, pointing toward a change in electronic state or ligand field around Zr, most plausibly driven by loss of terephthalate linkers and subsequent recoordination with biological ligands. The EXAFS data reinforce this interpretation. In pristine **UiO-66**, the Fourier transform magnitude of the EXAFS oscillations shows clear signatures of both the first coordination shell (Zr–O bonds at ∼1.7 Å) and the second shell (Zr–Zr and Zr–C correlations around ∼3 to 4 Å), consistent with the intact Zr_6_O_4_ node. After natural BHW exposure for 7 days, these features remain recognizable, albeit somewhat dampened, indicating only partial surface aging or possibly minor linker detachment. However, in the daphnid-exposed sample, the EXAFS oscillations are strongly attenuated, and the second coordination shell is nearly absent (Figures 1e,f). This collapse of medium-range order indicates that the crystalline Zr_6_ clusters no longer persist inside the organism, and instead Zr exists in a more amorphous, disordered form. Such behavior is characteristic of Zr species that have reprecipitated as oxides, hydroxides, or phosphate complexes - the latter being highly plausible given the phosphate-rich environment of the *D. magna* gut.^22^

The transformation observed here is thus distinct from abiotic aging. While BHW exposure alone leads to negligible structural perturbations, biological processing inside the daphnids *drives* a more severe and irreversible restructuring of the Zr coordination environment. This shift from an ordered Zr–carboxylate framework to amorphous Zr species demonstrates that **UiO-66** is not environmentally or biologically inert but instead undergoes pronounced *in vivo* transformations. Dissolution studies in both BHW and deionized water over 0–48 h revealed no detectable release of Zr from **UiO-66**. Consistent with this, an OECD TG 318–style suspension stability test (up to 6 h, varying pH, ionic strength and temperature) showed no measurable Zr ion leaching under any condition, indicating that the observed effects in *D. magna* are unlikely to be driven by dissolved Zr, but rather by *in vivo* biotic transformation of the **UiO-66** particles. Such outcomes highlight the necessity of incorporating biological exposure pathways into stability assessments of MOFs, since assessment of stability in abiotic media alone underestimates their susceptibility to breakdown in biological milieu.

TEM provides direct visual evidence of the loss of framework structure and morphology postdepuration in daphnids. Pristine **UiO-66** (Figure 1g) consists of uniform, well-faceted cubic crystals roughly 50–100 nm in size. After 7 days BHW exposure (Figure 1h), many particle edges appear slightly eroded or fused but still maintained cubic structure and crystallinity (Figure S1c). In the *daphnid*-exposed sample (Figure 1i), the loss of crystallinity is even more pronounced: at the 200 nm scale no intact crystals are visible, only clouds of aggregated nanoparticles. Even the largest clumps consist of agglomerated nanoscale particles. The TEM images confirm that ingestion of the MOF crystals by *D. magna* results in their transformation from crystalline MOFs into noncrystalline nanoscale particle agglomerates within 24 h.

Taken together, the XRF, XANES, and EXAFS results provide convergent evidence that **UiO-66** does not persist as an intact MOF inside *D. magna.* Instead, **UiO-66** is degraded within the digestive tract, releasing its organic linkers and transforming the Zr nodes into new, amorphous nanoparticle species. This finding has critical implications for both environmental safety and the potential long-term bioavailability of MOF-derived elements in aquatic systems. Crucially, the breakdown produces no new crystalline Zr phases (XANES shows no change); rather, the Zr likely ends up in amorphous or poorly ordered Zr–O clusters. From an environmental perspective, this means that released MOF particles will gradually collapse, releasing organic linkers and dispersing Zr (IV) species into the surroundings. Therefore, while **UiO-66** retains its expected Zr coordination under ideal conditions, under environmental and biological conditions, such as in the *D. magna* gut, the framework is progressively dismantled.

TEM/STEM-EDS provides direct, complementary evidence for the *in vivo* transformation of **UiO-66** inferred from X-ray spectroscopy. Pristine **UiO-66** presents well-faceted nanocrystals with spotty Selected area (electron) diffraction (SAED) patterns (Figure 2a), consistent with the intact Zr–carboxylate framework observed by bulk PXRD/ATR-FTIR and the preserved near-edge features in abiotic aging experiments (BHW, 7 d) (Figure 1d,e). In contrast, depurated material recovered after *D. magna* exposure exhibits rounded, fused aggregates with diffuse SAED rings and no resolvable lattice fringes (Figure 2b,c), evidencing loss of crystallinity within 24 h of ingestion. These morphological changes mirror the micro-XANES white-line suppression and EXAFS second-shell loss recorded inside the gut, which together indicate breakdown of ordered Zr_6_ nodes into disordered Zr–O environments.

**Figure 2:**
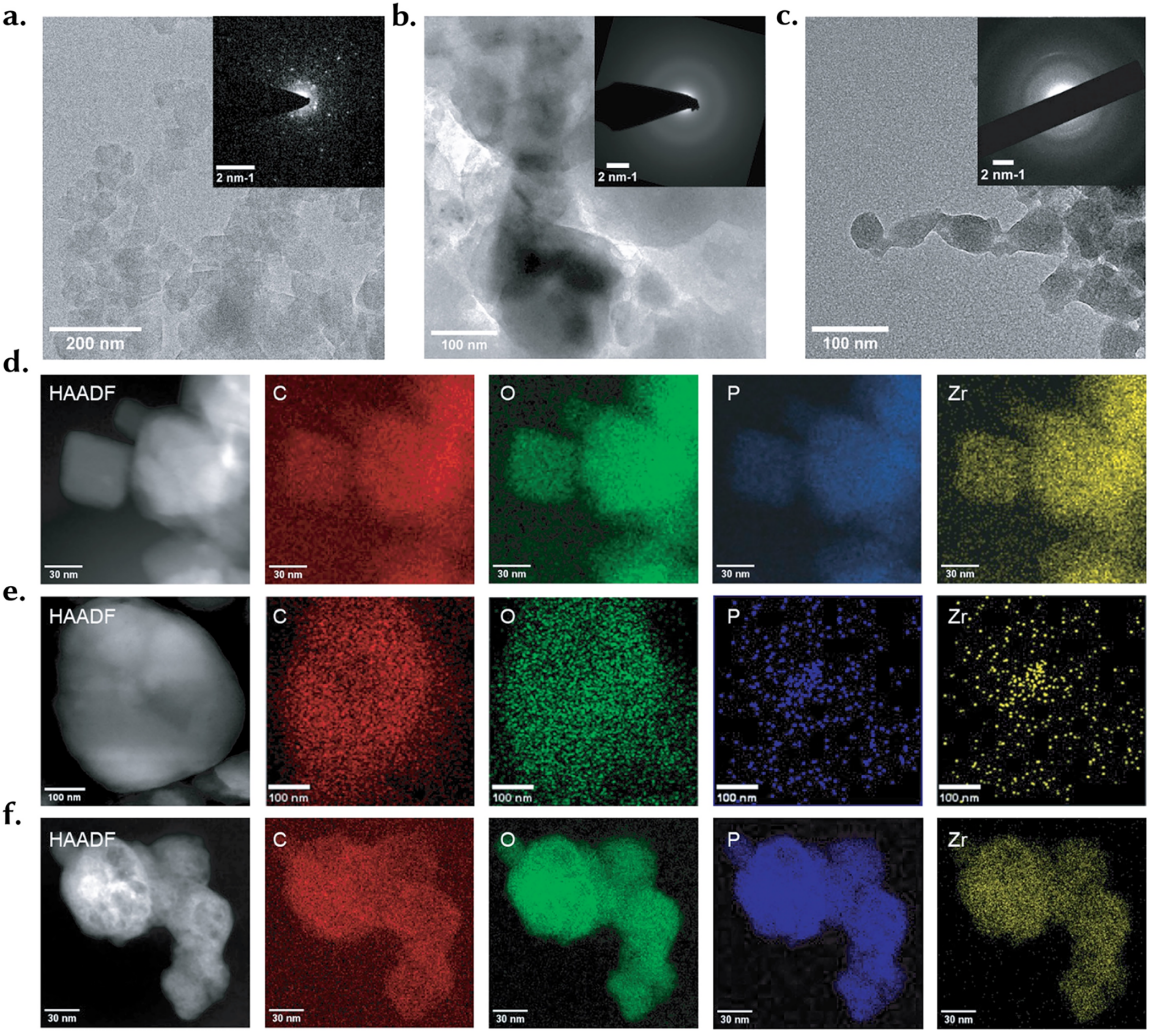
Biotic transformation mediated morphological collapse and element redistribution of UiO-66, resolved by TEM-SAED and high-angle annular dark-field scanning transmission electron microscopy (HAADF-STEM) and energy-dispersive X-ray spectroscopy (EDS) mapping. (a) Pristine **UiO-66** shows faceted nanocrystals with sharp edges (scale bar 200 nm); SAED inset displays discrete reflections, consistent with high crystallinity. (b) Depurated material after *D. magna* exposure at 10 μg mL^–1^ appears rounded and fused (scale 100 nm); the SAED inset shows diffuse rings indicative of amorphization. (c) Depurated material after exposure at 50 μg mL^–1^ forms aggregated, irregular nanodomains with loss of lattice order (scale 100 nm); the SAED inset shows pronounced diffused background and weak polycrystalline ring patterns. (d-f) HAADF-STEM images (left, scale bars 30–100 nm) with corresponding EDS maps for C (red), O (green), P (blue), and Zr (yellow) for pristine (d), 10 μg mL^–1^ depurated (e), and 50 μg mL^–1^ depurated (f) samples. Pristine particles display strong Zr colocated with the crystalline domains and negligible P. In depurated samples, P emerges and colocalizes with Zr, while Zr appears as punctate/clustered domains embedded in a carbon-rich organic matrix; colocalization is more extensive at 50 μg mL^–1^. The combined loss of crystallinity (TEM/SAED) and Zr–P colocalization (EDS) is consistent with biotic transformation of **UiO-66** into amorphous Zr–O/P species and redeposition within faecal matters.

Scanning transmission electron microscopy (STEM) with high-angle annular dark-field (HAADF) imaging coupled to energy-dispersive X-ray spectroscopy (EDS) mapping resolves the associated elemental redistribution (Figure 2d–f). Pristine crystals show Zr coregistered with particle interiors and negligible P. After gut passage, P appears and colocalizes with Zr, while Zr occurs as punctate nanodomains embedded in a carbon- and oxygen-rich matrix typical of faecal organic matter. The spatial concordance of Zr and P is stronger at 50 μg mL^–1^ than at 10 μg mL^–1^, suggesting greater extent of reprecipitation at higher body burdens. Corresponding EDS spectra (Figure S6) show the emergence of P Kα (∼2.01 keV) only in depurates, as confirmed by peak fitting, alongside strengthened Zr K lines; together with the XAS signature of amorphous Zr–O coordination, this pattern is most parsimoniously explained by ligand loss and recoordination to phosphate/hydroxide species^22,23^ in the phosphate-rich digestive milieu of *D. magna*. This interpretation is consistent with documented phosphorus chemistry in daphnid guts and with our micro-XAS evidence that postingestion Zr occupies a chemically uniform, disordered state across gut regions.

### 2.3 Implication of the Biotic Transformation of **UiO-66** for its Toxicity to *D. magna*

The acute toxicity of **UiO-66** toward *D. magna* was assessed by quantifying immobilization across exposure concentrations ranging from approximately 10 to 200 μg mL^–1^ at 24 and 48h (Figure 3a). No lethality was observed at concentrations up to 100 μg mL^–1^, confirming minimal acute toxicity at environmentally relevant doses. However, at the highest concentration tested (200 μg/mL), lethality increased to approximately 20%. Immobilization exhibited clear dose- and time-dependent trends, with immobilization rates at 24 h remaining below 60% even at the highest concentration, while sharply increasing by 48 h, surpassing 80% at concentrations exceeding 100 μg mL^–1^. Using the dose response eq (eq 1), the calculated EC_50_ value for **UiO-66** was determined to be 26.49 μg/mL. Interestingly, exposure to Zr ion and BDC (the organic linker) at an equivalent concentration showed <∼20% immobilization after 48h of acute exposure (Figure S5a,b) and is consistent with previously reported studies.^24^ Based on these acute toxicity findings, a sublethal concentration of 10 μg mL^–1^ (EC_10_ value) was selected for subsequent chronic toxicity experiments, ensuring minimal acute immobilization or lethality during prolonged exposures. Note of course that, as described in Section 2.2 above, the **UiO-66** particles were undergoing transformation in the daphnid gut during the exposure period.

**Figure 3:**
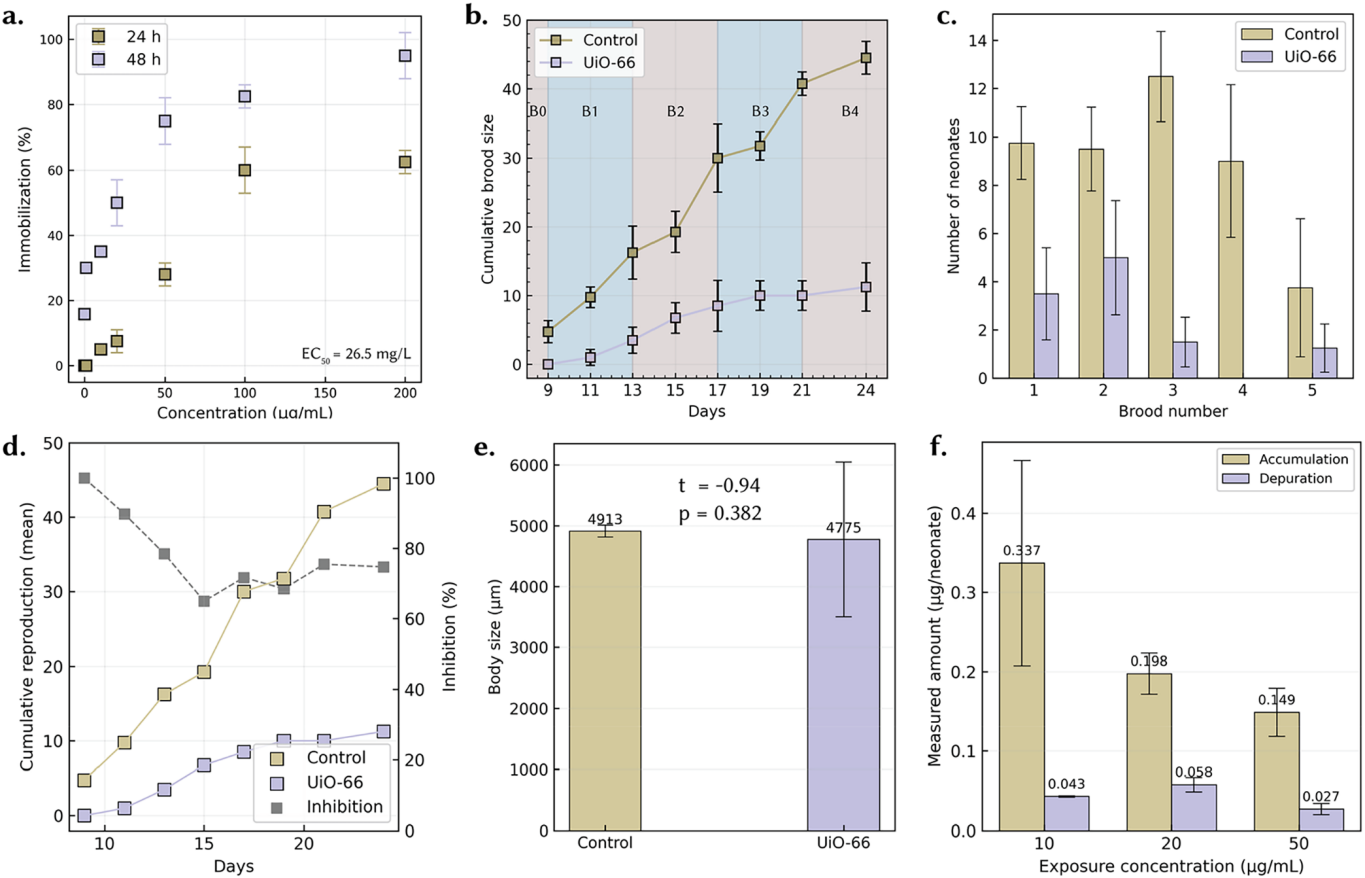
Ecotoxicological impacts of UiO-66 exposure on *D. magna.* (a) Acute immobilization as a function of concentration at 24 and 48 h in borehole water; a four-parameter sigmoidal fit using eq 1 gives EC_50_ ≈ 26.5 mg L^–1^, with minimal effects ≤100 μg mL^–1^. (b) Cumulative brood size over 24 days (brood windows B1–B4 shaded). **UiO-66** (10 μg mL^–1^; OECD-211) delays first reproduction by ∼3 to 5 days and maintains a persistent deficit relative to controls (differences highly significant from Day 13 onward). (c) Neonates per brood (B1–B5) showing consistent reductions in the treated group. (d) Cumulative reproduction (left axis) with percent inhibition relative to control (right axis), highlighting a sustained ∼70 to 75% inhibition after Day 15. (e) Body length of adults at test end shows no significant difference between groups (*t* = −0.94, *p* = 0.382), indicating reproductive effects were not driven by stunted growth. (f) Accumulation–depuration of Zr per neonate after 24 h exposure followed by 24 h in clean media at exposure concentration of **UiO-66** 10, 20, and 50 μg mL^–1^: accumulation consistently exceeds depuration (e.g., 0.337 vs 0.043 μg/neonate at 10 μg mL^–1^), evidencing substantial short-term retention and incomplete clearance. All data are mean ± SD (n shown in Methods).

Less than 24h third brood neonates of *D. magna* were chronically exposed to **UiO-66** at a concentration of 10 μg mL^–1^ for 21 days. At this sublethal concentration, no acute immobilization or mortality was observed during 48h tests - significant lethality only occurred at much higher concentrations (on the order of tens of μg/mL or above, based on acute assays). This indicates that **UiO-66** has a relatively low acute toxicity to *daphnids*, consistent with reports of low toxicity for ZrO_2_ nanoparticles (e.g., ZrO_2_ NPs showed 48h EC_50_ > 400 mg/L for *D. magna* immobilization).^25,26^ However, despite minimal short-term effects, chronic exposure to 10 μg mL^–1^ **UiO-66** for 21 days caused a clear reduction in reproductive output. Over the 21-day period, control *D. magna* produced a high number of offspring (cumulatively ∼44.5 ± 2.38 neonates per female by day 21), whereas **UiO-66**-exposed *D. magna* produced significantly fewer offspring (around 12.5 ± 4.14 neonates per female, roughly a 74% decrease in brood production relative to controls) (Figure 3b,c). Chronic exposure to **UiO-66** (10 μg mL^–1^) produced significant and sustained reproductive impairment over the 21-day life-cycle test. The reproduction timeline (Figure 3b) clearly shows that the cumulative brood size of the treated group consistently lagged behind that of the control from the onset of reproduction. Both groups showed similar early trends (days 9–11), but divergence became apparent by day 13, where control animals had produced ∼16 offspring compared to only ∼3 in the treated group. This difference widened rapidly in subsequent days, and by day 17 the control daphnids had produced ∼30 neonates versus ∼8.5 in the treated group. Statistical analysis (independent *t*-tests) confirmed that these differences were highly significant from day 13 onward (*p* < 0.0001).

Brood-wise analysis (Figure 3c) reinforces these findings, revealing substantial reductions in the number of neonates produced per brood. Control *D. magna* typically generated 9–13 neonates per brood, whereas **UiO-66** exposed animals rarely exceeded 4–5, and in some broods produced as few as 1–2 offspring. These reductions were consistent across all five reproductive cycles, suggesting that the impairment was not confined to a single brood event but represented a cumulative and persistent effect. Delayed brood initiation in **UiO-66** exposed organisms was also evident, with the first brood appearing 3 days later than in controls, indicative of slowed maturation and reproductive onset. The severity of reproductive suppression is further captured in the inhibition analysis (Figure 3d). Percent inhibition of reproduction remained high throughout the test period, starting above 90% in the early broods and stabilizing between 70–75% after day 15. This plateau suggests that **UiO-66** exposure induced a chronic stress state, preventing the recovery of reproductive performance even after acclimation. Importantly, the persistence of reproductive inhibition indicates that these effects are not transient or reversible within the time frame of the standard OECD 211 assay.

Accumulation–depuration dynamics corroborate a sustained internal burden consistent with the chronic effects. After 24 h exposure, neonates carried measurable Zr burdens that were only partially eliminated during a 24 h depuration in clean medium (Figure 3f). Mean 24 h clearance fractions were ∼13% (10 μg mL^–1^), 29% (20 μg mL^–1^) and 18% (50 μg mL^–1^), indicating substantial retention of transformed **UiO-66** particulates across doses. The nonmonotonic trend in accumulated mass (10 > 20 > 50 μg mL^–1^) likely reflects dose-dependent aggregation and settling that reduce suspended, ingestible particles and/or feeding suppression at higher loads. Together with the *in vivo* amorphization to disordered Zr–O/P phases evidenced by micro-XAS (Figure 1) and the postdepuration morphological collapse seen by TEM/EDS, these data imply that daily renewals in the 21-day test do not fully reset organismal burden. We therefore attribute the ∼ 74% reduction in reproduction at 10 μg mL^–1^ to persistent gut loading and energetic/physiological costs of handling and excreting poorly cleared precipitates rather than to growth inhibition, which was not observed (Figure 3e).

Visual observations of the organisms (Figure 4a,b) support the quantitative brood data. In the control group, the daphnids appear morphologically intact, with well-defined carapace edges, clear segmentation, and extended appendages. Their carapace is relatively transparent, allowing clear visualization of internal structures such as the gut, heart, and brood chamber. The brood chambers are prominently filled with developing embryos or eggs, indicating normal reproductive activity. Additionally, the gut appears uniformly filled with algae, suggesting consistent feeding behavior and healthy digestive function.

**Figure 4:**
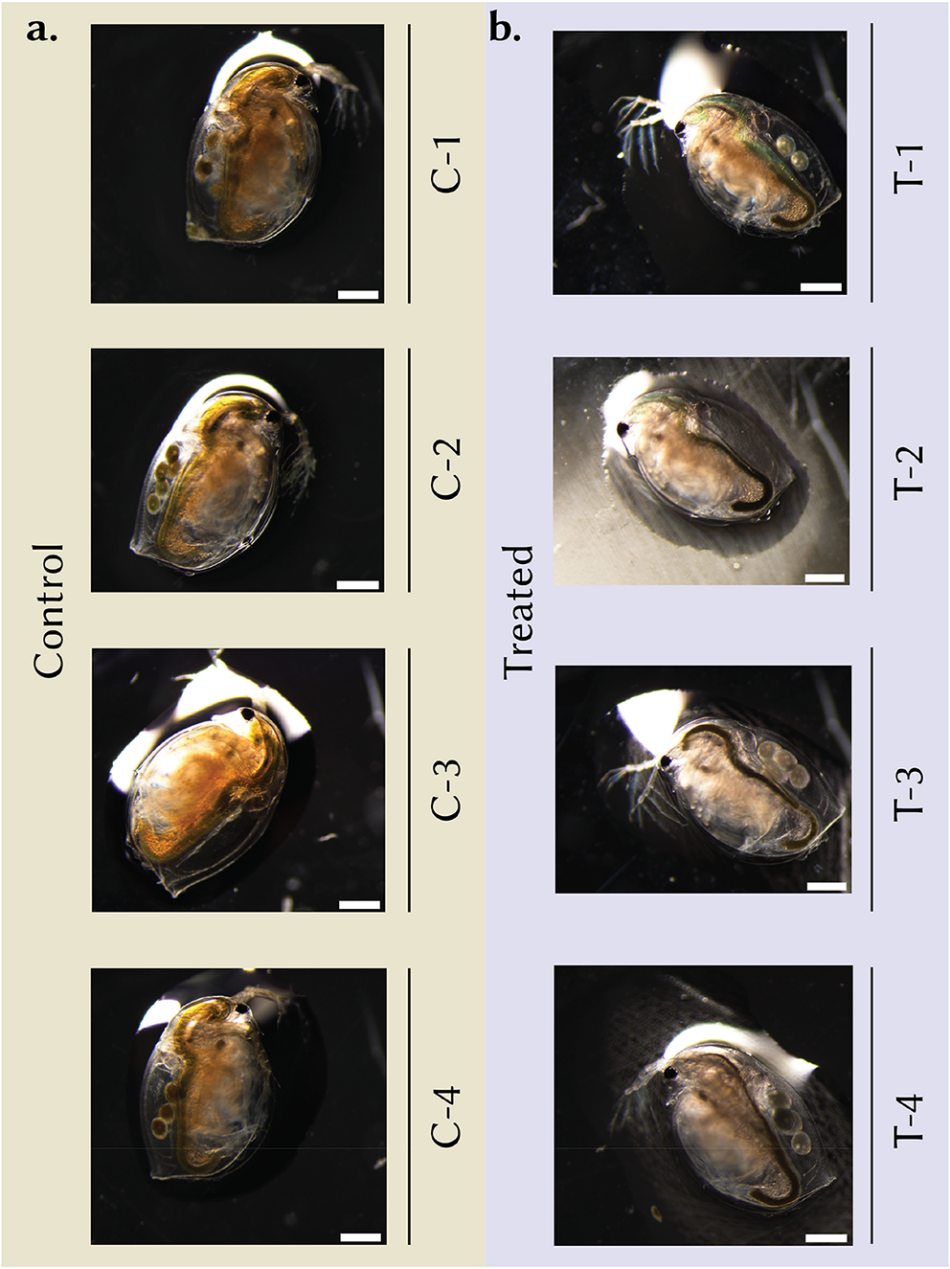
Representative optical images of *Daphnia magna* from the 21-day reproduction assay. (a) Controls (C-1–C-4) show normal brood development with brood chambers filled with developing embryos/eggs. (b) **UiO-66**–treated animals (T-1–T-4; 10 μg mL^–1^, OECD-211) exhibit reduced fecundity, with sparsely populated or empty brood chambers and occasional arrested embryos; darker gut contents are consistent with particle ingestion. Images were acquired at comparable magnification; scale bars as indicated. Body-length measurements for the same cohorts showed no significant difference between treatments, indicating that reproductive impairment was not driven by stunted growth (see accompanying analysis in Figure 3e).

In contrast, several individuals in the test group display subtle morphological alterations, including more irregular body outlines and slightly compressed postabdominal regions. The carapace appears opaquer in multiple individuals, which may be indicative of cuticular thickening, accumulation of particulate matter, or stress-induced pigmentation. Brood chambers are often less prominent or entirely empty, consistent with the significant reduction in reproduction observed in the chronic toxicity assays. Furthermore, test individuals exhibit darker or irregular gut regions, indicative of ingestion of the **UiO-66** particles (consistent with the XRF images in Figure 1a,b) and potentially altered feeding behavior. However, there was no statistically significant difference (p value = 0.382) between control and **UiO-66** exposed organisms in terms of their overall size (measured as eye-tail length) (Figure 3e), suggesting reproductive effects were not driven by stunted growth

The clear reduction in brood size and frequency is itself an ecologically significant outcome: even without causing acute mortality, the MOF exposure at 10 μg mL^–1^ over 21 days effectively lowered the reproductive rate of *D. magna*, which could translate to population-level impacts. In standard terms, one can consider the 21-day No Observed Effect Concentration (NOEC) for reproduction to be below 10 μg mL^–1^, since 10 μg mL^–1^ did cause a measurable effect on brood production in our test (though the exact threshold would require testing a range of concentrations). This finding aligns with prior observations that some nanomaterials have low acute toxicity but can significantly affect chronic end points like reproduction at much lower doses. For example, ZrO_2_ nanoparticles that were benign in 48h tests were found to inhibit *D. magna* reproduction by 50% at ∼96 mg/L (with a chronic NOEC of ∼0.8 mg/L), highlighting that prolonged exposure to even “non-toxic” materials can stress crustaceans and reduce their fecundity.^25^ In our case, 10 μg mL^–1^ (=0.01 mg/mL and 10 mg/L) of **UiO-66** over 21 days caused ∼74% reduction in reproduction, which is a substantial sublethal effect, though not as extreme as the 50% inhibition seen at higher doses in the ZrO_2_ nanoparticle study.^25^ Overall, the chronic toxicity of **UiO-66** to *D. magna* manifests as reproductive impairment and occurs at concentrations that produce no immediate lethality. Such outcomes reinforce the importance of long-term assays for novel/emerging materials like MOFs, as short-term tests alone would have missed these impacts on a critical fitness parameter.

Taken together, the colocalized Zr in the gut (Figure 1a,b), the uniform but altered XANES/EXAFS signatures (Figure 1c–f), and the postdepuration morphological collapse of the **UiO-66** structure (Figure 1i) demonstrate that **UiO-66** is rapidly (<24 h) biotically transformed *in vivo* into disordered Zr species that are not observed after abiotic aging in BHW. This *in vivo* transformation provides a mechanistic backdrop for the ∼74% suppression of reproduction and delayed brood timing quantified across Figure 3b–d. In practical terms, abiotic stability does not guarantee biological persistence: organism-specific microenvironments can remake even “robust” **UiO-66** MOFs into new Zr phases with distinct bioavailability and energetic costs (e.g., handling/excretion), thereby depressing reproductive performance without acute lethality. This underlines the need to integrate determination of biotic transformation pathways and their impacts into environmental fate and risk assessment of MOFs.

The structural and chemical transformation of **UiO-66** have two nonmutually exclusive consequences for organismal performance and environmental fate. First, insoluble Zr-rich precipitates forming *in situ* can accumulate within the gut, creating physical interference (blockage, false satiation) that reduces feeding efficiency and energy intake, consistent with the 3–5-day delay in brood initiation and ∼ 74% reduction in cumulative reproduction at 10 μg mL^–1^ despite negligible acute immobilization (OECD 211 vs OECD 202). Second, excreted aggregates are packaged into dense faecal pellets, promoting grazer-induced sedimentation that lowers pelagic exposure but redistributes transformed material to benthic habitats, where biocorona-laden fragments and liberated linkers may pose microbial or oxidative stress hazards. These outcomes align with our whole-organism mapping (Zr confined to gut), the *in vivo* amorphization evidenced by XANES/EXAFS, and the postdepuration collapse visualized by TEM. Together, they reinforce the central principle of this study – abiotic stability ≠ biological inertness and argue that mechanistic, *in vivo* transformation pathways must be integrated alongside chronic end points in environmental risk assessment of SSbD MOFs for water applications.^27,28^

Our observations point to a mode of action that is fundamentally different from classical dissolved-metal toxicity. **UiO-66** remains structurally intact in BHW, but once ingested it is rapidly converted into amorphous, phosphate-rich Zr phases that are spatially confined to the gut and only partially cleared during depuration. This creates a scenario where the daphnids are repeatedly exposed not to pristine MOF crystals but to poorly cleared, inorganic precipitates embedded in faecal and gut organic matter. The absence of any detectable reduction in body length, combined with a pronounced delay in first brood and a ∼ 74% suppression of cumulative reproduction at 10 μg mL^–1^, suggests that energetic trade-offs – for example, between maintenance/handling of gut loads and investment in reproduction – are more important than overt cytotoxicity or systemic Zr poisoning. Similar decoupling between gut-localized accumulation, limited depuration and marked sublethal effects has been reported for other inorganic nanomaterials in *D. magna*, including ZnO nanoparticles that remain along the digestive tract with negligible expulsion over 24 h and show size-dependent toxicity,^29^ and Ag nanoparticles that cause persistent physiological and metabolomic disturbances even after exposure ceases, largely attributed to silver retention and damage to the digestive epithelium.^30^ In ecological terms, such sublethal but persistent reproductive stress at concentrations that do not cause acute immobilization is likely to be more relevant for long-term population dynamics than short-term mortality end points. At the same time, the packaging of transformed **UiO-66** into dense faecal pellets implies that grazers can act as both transformers and vectors, shuttling “spent” MOF material from the pelagic zone into sediments. This dual role highlights that biotic transformation can simultaneously mitigate water-column exposure while creating new exposure pathways for benthic communities and sediment-dwelling microbiota, reinforcing the need to consider whole-ecosystem redistribution when evaluating the safety of nominally stable MOFs.

Beyond **UiO-66**, there is growing evidence that MOF type and, in particular, the nature of the metal center and framework stability strongly influence toxicity. Cu-based MOFs such as **HKUST-1** are hydrolytically labile in aqueous media and readily release Cu^2+^, which is well-known to be highly toxic to aquatic organisms. For example, nanoscale **HKUST-1** causes marked acute toxicity in zebrafish, with 96 h LC_50_ values of ∼1.5 to 2.1 mg L^–1^ in embryos and adults, and toxicity correlates with Cu^2+^ release and bioaccumulation in tissues.^31^ In freshwater algae, **HKUST-1** induces pronounced growth inhibition, oxidative stress and transcriptomic perturbations, whereas a more stable carbonated derivative (**DHKUST-1**) exhibits reduced Cu^2+^ leaching and significantly lower toxicity.^32^ Similar patterns are observed for Zn-based MOFs such as **ZIF-8**:^33^ partial dissolution and Zn^2+^ release in water have been linked to developmental malformations, cardio-behavioral defects and neurobehavioral alterations in zebrafish embryos and larvae, as well as oxidative stress and tissue damage in benthic bivalves such as *Corbicula fluminea*.^34^ A recent comparative study across five MOF nanoparticles in adult zebrafish found a clear toxicity ranking of Cu-MOF > **ZIF-90** > **ZIF-8** > Fe-MOF > Zr-MOF, highlighting that both metal identity and framework stability are critical determinants of hazard.^35^ Our work on Zr-based **UiO-66** therefore complements this literature by showing that even a comparatively water-stable, low-solubility MOF can cause significant chronic ecotoxicity once biotically transformed *in vivo*, while less stable Cu- and Zn-based MOFs may pose additional risks through metal-ion release and associated aquatic toxicity.

### 2.4 Implications for Stakeholder in MOF Development and Applications

While **UiO-66** is renowned for its chemical robustness, and it remains highly stable in natural borehole water, the biological milieu of *D. magna* completely alters its structural identity. Using microfocus XAS in a landmark application of this method at such a fine spatial scale, we tracked **UiO-66**’s fate *in vivo* and linked the biotic transformation to negative impaired reproductive outcomes. This study thus, redefines how nanoscale material transformations should be studied: capturing *in vivo* structural alterations and correlating these with organism-level responses.^36^ Notably, after ingestion, passage through the gut and excretion (depuration into clean medium), **UiO-66** particles are encapsulated within dense faecal pellets. Such heteroaggregation precipitates the material from suspension, effectively removing it from the water column - an example of grazer-induced sedimentation. While this may reduce (further) exposure for planktonic species, it simultaneously transfers transformed materials into sediments, raising concerns about benthic exposure and ecosystem redistribution.

Inside the *D. magna* gut, **UiO-66** loses its ordered Zr–carboxylate clusters and transforms into amorphous Zr phases, likely including hydroxides and phosphates, driven by enzymatic activity, acidic pH, and phosphate-rich conditions inherent to the daphnid gut. These transformations were spatially localized to the gut (Figure 1a), uniform across gut regions, and confirmed by dampened XANES white-line features and collapse of EXAFS second shell peaks. This demonstrates that the intrinsically robust MOFs are reconfigured *in vivo* into fundamentally different compounds. The transformation of **UiO-66** to insoluble Zr species may have dual consequences. On one hand, reduced solubility lowers chemical bioavailability and thus the risk of metal-driven toxicity. However, because this transformation occurs after ingestion, the resulting insoluble precipitates may accumulate in the gut, potentially blocking passage or creating a false sense of satiation. Such physical impacts could impair feeding efficiency, reduce calorific intake, and ultimately delay sexual maturity in *daphnid*. Sequestration via faecal aggregation may lower exposure risk for pelagic organisms. On the other hand, the release of terephthalate linkers or biocorona-laden fragments may disrupt microbial communities or induce oxidative stress. Because these transformations occur within the *D. magna* gut, the gut epithelium itself may be an immediate site of toxic action, before fragments are excreted and enter benthic environments where they could create additional hazards.

This biotic transformation aligns with our reproductive toxicity data: a ∼ 74% decline in neonate output over 21 days at only 10 μg mL^–1^ **UiO-66** - despite no acute immobilization. We posit that gut-level processing and MOF breakdown inflict physical interference, physiological stress, and energetic burdens that compromise reproduction. Ultimately, the implications are profound for MOF developers and MOF-applications. Abiotic stability in natural water does not guarantee biological inertness. Organism-level interactions, not just chemical properties dictate fate and transformations. Integrating microfocus XAS spatial mapping with ecotoxicological end points offers a powerful means to trace these changes *in situ*. Our results highlight that

1. Biotic interactions can significantly mitigate (through sediment removal) or amplify (through altered reactivity) nanomaterial toxicity.
2. *In vivo* transformation pathways must be incorporated into environmental risk assessments even where acute tests indicate no toxicity.
3. Chronic, sublethal end points such as reproduction are already a core requirement in regulatory ecotoxicology. However, our findings emphasize that these end points alone may not be sufficient. They need to be complemented by mechanistic investigations-such as tracking biotic transformation pathways, gut-level interactions, and energy trade-offs – to uncover why reproduction is impaired. Without integrating such mechanistic understanding, the hidden risks of ‘stable’ MOFs may remain underestimated, even when standard chronic assays are performed.

This study (Figure 5) thus, in our opinion sets a new precedent: tracing *in situ* transformation of a nominally ’stable’ MOF in a living organism and demonstrating its consequences for the organism and the MOF. In an age when MOFs are increasingly considered for deployment for remediation and industrial use, such insights are vital to ensuring that deployment strategies are genuinely sustainable and ecologically protective and consider the whole life cycle of the material. While we focus on the transformation mechanism and chronic reproductive outcomes, our work opens avenues for further research into the specific fate of the organic linkers and the long-term ecosystem-level impacts of benthic transfer.

**Figure 5:**
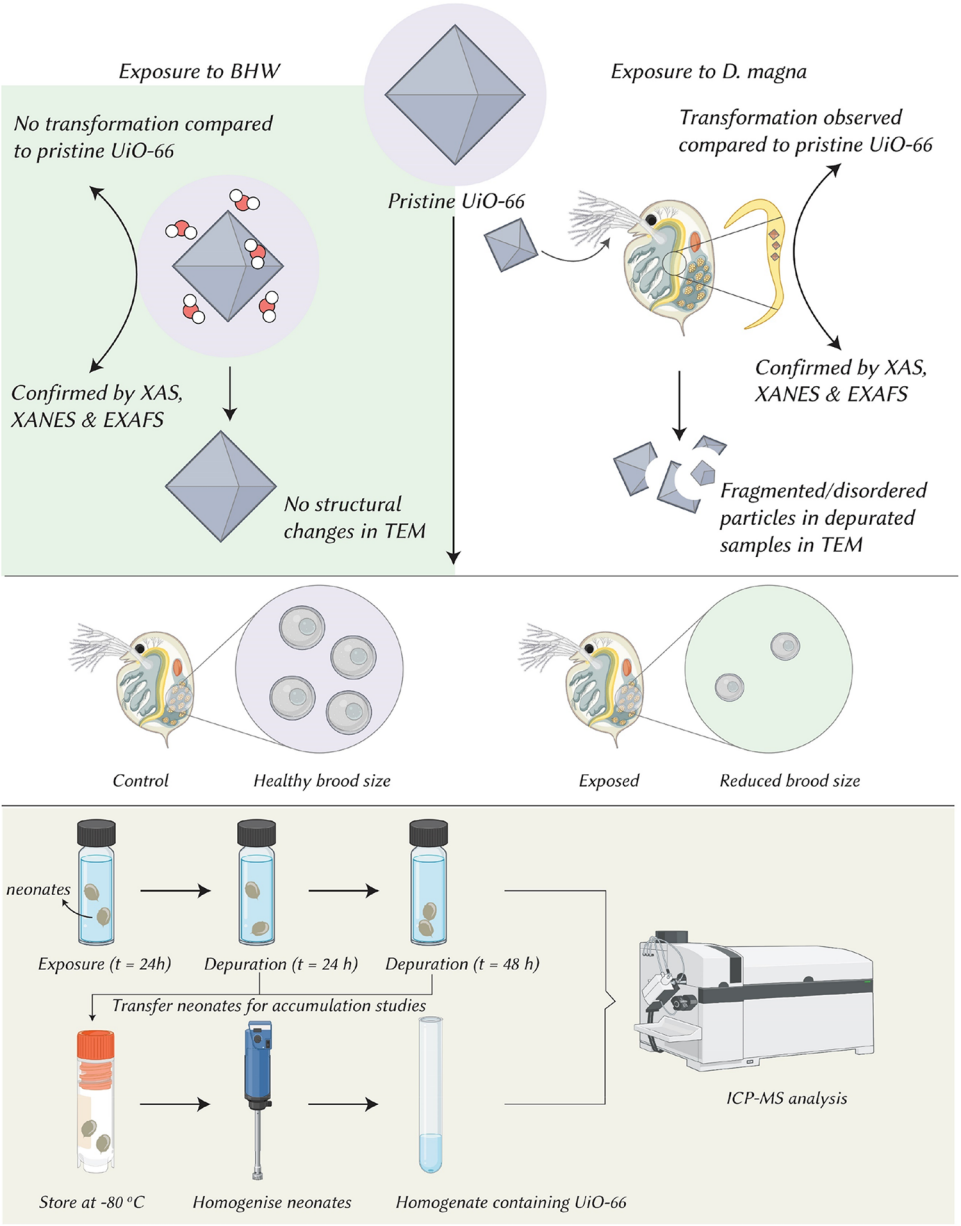
The storyline. UiO-66 is abiotically stable in borehole water, but after ingestion by *D. magna* it transforms *in vivo*: micro-XANES/EXAFS indicate collapse of the Zr–carboxylate framework and TEM of depurates shows fragmented, disordered particles. This gut-level transformation coincides with sublethal yet severe reproductive impacts – delayed first brood and reduced brood size at 10 μg mL^–1^ despite minimal acute effects. An accumulation–depuration workflow (24 h exposure → 24–48 h depuration → homogenization/ICP-MS) reveals substantial short-term retention and incomplete clearance, linking transformation chemistry to organismal outcomes. *D. magna* illustration sourced from Vecteezy.com.

## 3 Conclusions

Here, we show that nanoscale **UiO-66**, although structurally robust in natural BHW, undergoes rapid biotic transformation in the gut of *D. magna*. Microfocus XRF, XAS, and TEM revealed that ingested **UiO-66** loses its long-range order and converts into amorphous, phosphate-rich Zr phases that are spatially confined to the gut and faecal material. These structural changes occur on ecologically relevant time scales and are not apparent from abiotic aging alone, highlighting the importance of *in vivo* transformation pathways for understanding the fate of MOFs in aquatic organisms. Chronic exposure to environmentally realistic **UiO-66** concentrations led to marked sublethal effects, including delayed onset of reproduction and substantial suppression of cumulative brood production, despite limited acute immobilization. These findings indicate that the main biological burden arises not from pristine MOF particles or dissolved Zr, but from poorly cleared transformation products and associated energetic and physiological costs. Consequently, abiotic stability in water cannot be taken as a proxy for biological inertness, particularly for materials intended for use in water-treatment applications where direct contact with aquatic biota is likely. More broadly, this work highlights the need to integrate transformation-aware testing strategies, chronic end points, and high-resolution structural probes into the environmental assessment of MOFs and other advanced materials. Considering how materials evolve within organisms, and not only in simplified abiotic media, will be critical for designing SSbDMOFs whose performance benefits do not come at the expense of long-term ecological health.

## 4 Experimental Section

### 4.1 Materials

All reagents used for the synthesis of **UiO-66** were of analytical grade. The freshwater crustacean *D. magna* (BHAM-2 strain) was used for biotic exposure and ecotoxicity studies.

### 4.2 Synthesis, Characterization and Stability of **UiO-66** MOFs

Nanosized **UiO-66** was synthesized via a conventional solvothermal procedure with slight modifications.^37,38^ Zirconium chloride (ZrCl_4_, 0.6435 mmol, ∼0.150 g) and benzene-1,4-dicarboxylic acid (BDC, 0.6435 mmol, ∼0.107 g) were first dissolved in 75 mL of N, N-dimethylformamide (DMF) with continuous stirring for 30 min to ensure complete dissolution and molecular-level homogeneity which is critical for controlled nucleation of **UiO-66**. The clear, homogeneous mixture was then transferred to a Teflon-lined autoclave and heated to 120 °C for 24 h using solvothermal method. Upon cooling back to room temperature, the white crystalline precipitate was collected via centrifugation, rinsed multiple times with ultrapure water to eliminate unreacted precursors and residual solvent, and subsequently activated by overnight vacuum drying at 120 °C. The synthesized powdered samples underwent rigorous structural and morphological characterization. Powder X-ray diffraction (PXRD, Malvern Panalytical, UK), operating with a Zr–Kα source (40 kV, 30 mA) over a scanning range of 5–50° 2θ, confirmed the expected face-centered cubic (fcc) crystal structure and high crystallinity. Morphological inspection via SEM (JEOL JSM-7900F) and TEM (JEOL 1400, Japan) revealed uniform, octahedral **UiO-66** crystals. For TEM analysis, the samples were dispersed on ultrathin carbon films supported by 200-mesh gold grids, enabling high-resolution particle imaging. Diffraction and composition analysis were performed on the Talos F200X G2 TEM and Tecnai TF20, both operated at 200 kV electron voltage. SAED analysis was performed under low-dose exposure to avoid beam-induced damage which is inherent for the **UiO-66**.^39^ The beam current, measured on the samples, was kept at 0.074 nA in both microscopes using the lowest condenser aperture (10 μm) and numerical number 6 of spot size. STEM and EDS were performed to produce the distribution map of all presenting elements. STEM-EDS mapping was performed with the probe current of 1.2 nA (measured outside the sample), convergent semiangle of 10 mrad, and the dwell time of 50 μs. To probe the local zirconium coordination environment, Zr K-edge X-ray absorption spectroscopy (XAS) measurements were carried out at the B18 Core EXAFS beamline of Diamond Light Source (experiment IDs: MG33674–1, SP35117–1, SP35776–1). This involved Quick-EXAFS (QEXAFS) scanning in transmission mode using a Pt-coated optics system, with an Si (111) monochromator and zirconium foil standard for energy calibration. Room-temperature data were collected with each sample scanned three times to improve signal-to-noise ratio; data reduction used the Demeter suite to ensure robustness and reproducibility.

To perform a stability test based on OECD 318 guidelines,^40^ **UiO-66** stock dispersions were prepared by calibrated probe sonication in ultrapure water and diluted to ∼10^12^ particles L^–1^. Screening tests were performed in triplicate at pH 4, 7, 9 (±0.2) with Ca­(NO_3_)_2_ = 0, 1, 10 mM at room temperature and 37 °C. The pH was stabilized with 5 mM NaHCO_3_ as recommended by TG 318. Aliquots (0.5 mL) were withdrawn from the top 0.5–1 cm at 0 and 6 h; immediately before the 6 h sampling, tubes were centrifuged under conditions equivalent to a ∼1 μm hydrodynamic cutoff (per OECD calculation). Zr was quantified by ICP-MS (NexION 350, PerkinElmer, USA) using a standard curve from 1 to 10,000 μg mL^–1^ prepared in 0.1 M ammonium bicarbonate, analyzed in helium KED mode (He gas flow 4 L min^–1^); samples were matrix-matched and diluted as needed. Where relevant, total Zr was complemented by <10 kDa ultrafiltrate to estimate dissolved Zr. Stability (%) was calculated as C_6_h/C_0_h × 100 (C_0_h set to 100%), and classified as low (≤10%), condition-dependent (10–<90%), or high (≥90%) per TG 318.

### 4.3 Abiotic and Biotic Transformation Studies

To study transformation under environmental conditions, **UiO-66** particles were aged in natural borehole water (BHW, University of Birmingham, Table S1) by suspending 100 mg of material in 100 mL of test solution (1000 mg L^–1^) with gentle stirring over 7 days. Postaging, particles were recovered by centrifugation (8000 rpm, 10 min), washed with deionized water, and air-dried. These aged samples were characterized via FTIR (Spectrum2, PerkinElmer) and XPS for surface chemical changes, TEM for morphological alterations, PXRD for structural integrity, and Zr K-edge XAS (mounted in Kapton washers, measured in ambient conditions) to assess changes in local coordination.

Finally, to capture biotic transformation, *D. magna*, third brood neonates was exposed to **UiO-66** dispersions at 100 mg L^–1^ in BHW for 24 h under controlled ecotoxicological conditions. Postexposure, daphnids were rinsed to remove surface-bound MOF, lyophilized to preserve internal nanoparticle distribution, and subjected to microscale XRF mapping and micro-XAS at Diamond Light Source I18 beamline. Another set of **UiO-66** exposed individuals were placed into fresh BHW for 24 h to allow faecal pellet egestion (depuration); these pellets, which contained excreted UiO-66, were collected and imaged using TEM to visualize gut-mediated morphological transformation.

The micro-XRF mapping and XAS measurements on *D. magna* were conducted at the microfocus I18 beamline^41,42^ of the Diamond Light Source (experiment ID: SP40942–1). The lyophilized daphnid (mounted on Kapton tape) was placed at a 45° geometry to the incident X-ray beam. A finely focused hard X-ray beam (∼2.5 μm spot size) was used to raster-scan the organism and produce XRF elemental maps, highlighting the distribution of Zr and other elements within the tissue. After mapping, XAS spectra (primarily XANES) at the Zr K-edge were collected at selected regions of interest in the daphnid (e.g., gut area or appendages where UiO-66 accumulated). The I18 beamline uses an Si (111) monochromator for energy selection, similar to B18, ensuring a narrow-band incident beam on the sample.^43^ XAS data on the daphnid were collected in transmission mode (due to high uptake of Zr by the organism).

XAS data (both bulk and microfocus) were processed using the Demeter software package (Athena/Artemis).^44^ The three scans for each sample were aligned (using the Zr foil as reference), averaged, and background-subtracted to extract the extended X-ray absorption fine structure (EXAFS). Fourier transforms of Zr K-edge EXAFS spectra were then generated to analyze changes in the Zr coordination environment across different conditions. XRF spectral data and elemental maps from the *D. magna* experiments were analyzed with PyMCA software for quantification of elemental signals.

### 4.4 Ecotoxicity Testing of **UiO-66**

#### 4.4.1 Acute Immobilization Study

The acute toxicity of **UiO-66** to *D. magna* was assessed using a 48h immobilization assay based on OECD Test Guideline 202,^12^ with slight modifications to reflect environmentally realistic conditions. Neonates of *D. magna* (<24 h old) were obtained from healthy laboratory-maintained cultures of Bham-2 strain. Each test vessel contained 5 neonates in 10 mL of BHW, which was selected as the test medium to replicate the conditions used in prior aging studies of **UiO-66**. Exposure groups included a range of **UiO-66** concentrations (0, 10, 25, 50, 100, 150, and 200 μg/mL), prepared freshly in BHW for each test, and distributed across four replicate vessels per concentration. This design yielded 20 daphnids per concentration per experiment, and the entire experiment was conducted in triplicate (*N* = 3) for reproducibility.

Tests were performed in a temperature-controlled incubator maintained at 20 ± 1 °C under a 16:8-h light: dark photoperiod, with no feeding during the test period, as per guideline recommendations. Immobilization was recorded at 24 and 48h by observing whether daphnids were capable of swimming within 15 s after gentle agitation of the test vessel. The negative control group (BHW only) showed ≤ 10% immobilization, confirming test validity. Dose response data from all three experiments were pooled and modeled using a four-parameter sigmoidal logistic regression to calculate the 48h EC_50_ value. The dose–response equation used was

1 $$Y=A1+\frac{A2-A1}{\left(1+10\left(\left(\log\;X0-X\right)\times p\right)\right)}$$

where *A*1 and *A*2 are the minimum and maximum responses, *x* is the log concentration, Log X0 is the inflection point (log EC_50_), and *p* is the Hill slope. An EC_10_ value was also calculated for use in the chronic tests.

#### 4.4.2 Chronic Toxicity Testing

To evaluate long-term sublethal effects, a 21-day chronic toxicity assay was performed following OECD Test Guideline^13^ 211, with the key modification of using natural BHW instead of synthetic media to ensure environmental realism. Neonates (<24 h) were individually placed into glass beakers containing 50 mL of either control (BHW only) or treated medium (BHW + 10 μg/mL **UiO-66**). The selected exposure concentration of 10 μg/mL was selected as the EC_10_ from the acute assay and represented a sublethal dose. Each treatment group consisted of four replicate vessels per experiment, with the full assay repeated over two independent experiments (*n* = 8 total per treatment group).

Daphnids were maintained at 20 ± 1 °C under a 16 h light/8 h dark cycle and were fed every 2 days with a standard freshwater algal suspension at guideline-recommended levels (approximately 0.1–0.2 mg C/daphnid/feed). Every 2 days, coinciding with feeding, the test medium was gently replaced to maintain consistent **UiO-66** exposure levels and water quality. During renewal, adult daphnids were transferred to fresh medium using wide-bore pipettes to prevent mechanical stress. Key end points included adult survival, reproductive output (cumulative brood size), and time to first brood. Offspring were counted and removed during each medium renewal to avoid resource competition. Brood size was tracked separately for each brood (B1–B4), allowing assessment of whether longer exposure to **UiO-66** led to increasingly severe impacts on brood size. At the end of the 21-day exposure, adult daphnids were examined under a stereomicroscope to assess body length and identify any visible anatomical abnormalities. Representative individuals from both control and treated groups were imaged on Day 24. Independent two-sample *t*-tests, conducted using OriginPro software, confirmed statistically significant differences in brood size (*p* < 0.0001) from Day 13 onward. All experiments were performed in quadruplicate to obtain a statistically significant data.

#### 4.4.3 Accumulation–Depuration Assay of UiO-66

Accumulation and depuration studies were conducted using *Daphnia magna* neonates (<24 h old, Bham-2 strain). Test organisms were exposed to three nominal concentrations of **UiO-66** (10, 20, and 50 μg/mL) in BHW under static conditions. Experiments were performed in triplicate using acid-cleaned glass vials, each containing 10 neonates in 5 mL of test suspension (containing **UiO-66** in BHW). The exposure period lasted 24 h, during which no food was provided to avoid confounding interactions with algae. Following exposure, neonates were carefully rinsed three times with fresh BHW to remove loosely bound particles and transferred into fresh BHW for depuration. The depuration phase was carried out for 24 h under identical conditions. At the end of the depuration, neonates were collected and frozen at −80 °C until further processing. Exposure and depuration media were retained for chemical analysis and stored at 4 °C until digestion and ICP–MS quantification. TEM was performed on a Tecnai F20 (Thermo Fisher Scientific) operated at 200 kV. Pristine **UiO-66** and depurated materials recovered after *Daphnia* exposure at 10 and 50 μg mL^–1^ were drop-cast onto ultrathin continuous carbon films on 200-mesh gold grids and air-dried. Because **UiO-66** is beam-sensitive,^45^ SAED for pristine and depurated samples was acquired under low-dose conditions using the smallest condenser aperture (10 μm) and a high spot number (6), giving an on-sample beam current ∼0.074 nA. For composition and spatial distribution, the instrument was operated in STEM with HAADF imaging coupled to EDS to map C, O, P, and Zr. STEM–EDS maps were collected with a probe current ∼1.2 nA (measured upstream of the specimen), convergence semiangle 10 mrad, and dwell time 100 μs per pixel.

Exposure and depuration media were mixed by repeated pipetting prior to digestion. Aliquots (2.5 mL) were mixed with an equal volume (2.5 mL) of 1 M ammonium bicarbonate and digested following a previously reported two-step method with minor modifications.^46^ To facilitate complete breakdown of **UiO-66** structures, the suspensions were sonicated for 20 min in an ice bath. This adaptation was introduced to minimize thermal degradation of dissolved organic matter and to enhance digestion efficiency. The digests were then passed through 0.22 μm PTFE syringe filters to remove residual carapace fragments, undigested particulates, and organic debris, thereby preventing nebulizer clogging during ICP–MS analysis. **For accumulation studies**, frozen neonates from each treatment group were thawed on ice and homogenized in 2.5 mL of 1 M ammonium bicarbonate using a homogenizer for ∼10 s. An additional 2.5 mL of ultrapure water was added, and the suspensions were sonicated in an ice bath for 20 min to achieve complete digestion of UiO-66. The resulting digests were filtered through 0.22 μm PTFE syringe filters as described above and stored at 4 °C prior to ICP–MS analysis.

Zr concentrations were quantified using an ICP–MS (Nexion 350, PerkinElmer, USA), operated in KED mode with helium as the collision gas to minimize polyatomic interferences. Calibration standards were prepared from a certified zirconium standard solution (1000 μg/mL, Sigma-Aldrich, UK), diluted in deionized water. Internal standards (yttrium) were used to correct for matrix effects and instrumental drift. Procedural blanks and quality control standards were included every 10 samples to verify instrument stability and digestion reproducibility.

## Supplementary Material



## Notes

Additional experimental and analytical details and eight supplementary figures (Figures S1–S8) and one supplementary table (Table S1). Figure S1 shows physicochemical characterization of **UiO-66** before and after aging in BHW (TEM, PXRD, SEM, ATR-FTIR). Figure S2 presents high-resolution XPS of **UiO-66** before and after 7-day aging in BHW. Figures S3 and S4 compare Zr K-edge XANES and EXAFS spectra of **UiO-66** with ZrO_2_ and between Diamond beamlines (I18 and B18). Figure S5 reports acute immobilization of *D. magna* by dissolved Zr and the BDC linker. Figure S6 shows STEM–EDS spectra of pristine and depurated **UiO-66**, while Figure S7 provides micro-XRF maps of *D. magna* highlighting Zr and Ca distributions following exposure. Figure S8 summarizes OECD TG 318 suspension stability of **UiO-66** as a function of pH, ionic strength and temperature. Table S1 reports the composition, physicochemical properties and environmental relevance of the BHW) used in the exposure experiments (PDF)
