# Supplementary material for: Biotic Transformation of Abiotically Stable Nanoscale UiO-66 Metal–Organic Framework by Daphnia magna Results in Chronic Reproductive Toxicity
Source: ACS Nano. 2025 Dec 3;19(49):41874–87. doi: 10.1021/acsnano.5c16532 (PMC12774330; doi:10.1021/acsnano.5c16532)
Supplement: Supplementary file 1 [file nn5c16532_si_001.pdf]

Supplementary Information for

Biotic Transformation of abiotically stable nanoscale UiO-66 Metal–Organic Framework by *Daphnia magna* results in Chronic Reproductive Toxicity

Swaroop Chakraborty[a,b,!]*, Pankti Dhumal[a,b,!], Iuliia Mikulska[c], Sang Pham[d], Laura-Jayne Ellis (Bradford)[a,b], Dhruv Menon[e], Superb K. Misra[f], Iseult Lynch[a,b]

[a] School of Geography, Earth & Environmental Sciences, University of Birmingham, Edgbaston, B15 2TT, UK

[b] Centre for Environmental Research and Justice (CERJ), University of Birmingham, Edgbaston, B15 2TT, UK

[c] Diamond Light Source, Harwell Science and Innovation Campus, Didcot, OX11 0DE UK.

[d] Facility of Electron Microscopy, University of Birmingham, Edgbaston, B15 2TT, UK

[e] Department of Chemical Engineering & Biotechnology, University of Cambridge, Cambridge, CB3 0AS, UK

[f] Materials Engineering, Indian Institute of Technology, Gandhinagar, 382355, India.

*Email- s.chakraborty@bham.ac.uk

[!] Authors with equal contribution

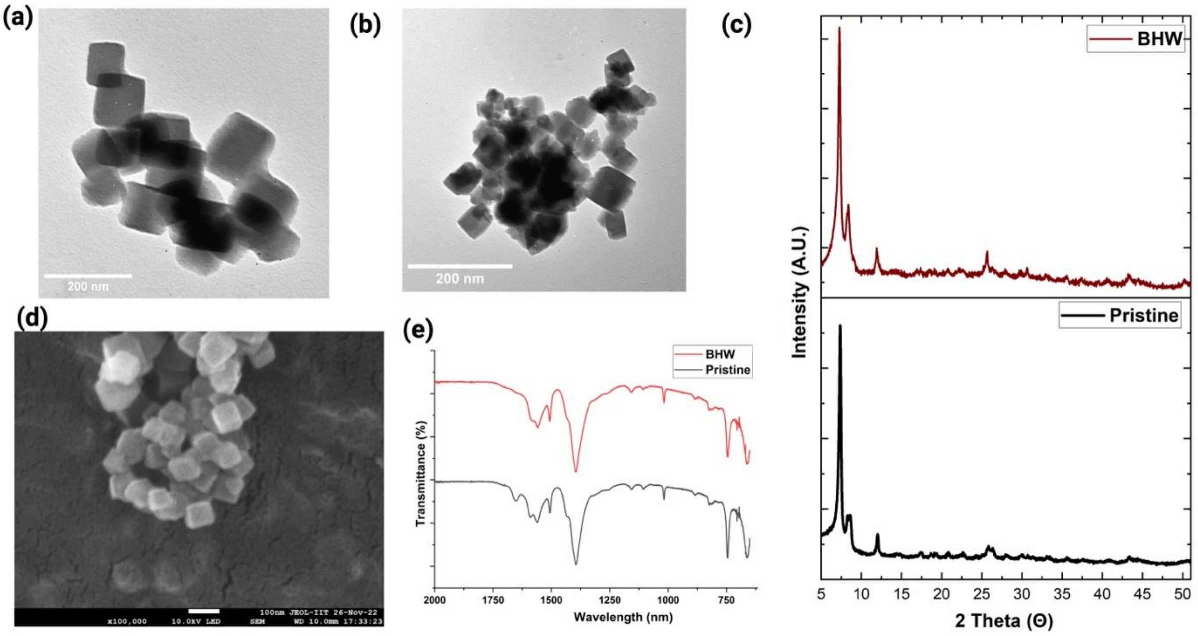

Figure S1. Physicochemical characterisation of UiO-66 before and after ageing in BHW. (a) TEM of pristine **UiO-66** showing uniform, faceted octahedral crystallites (scale bar = 200 nm). (b) TEM of **UiO-66** after 7 days in BHW, retaining overall polyhedral morphology with mild agglomeration/surface roughening. (c) PXRD patterns of pristine (black) and BHW-aged (red) **UiO-66**: reflections characteristic of the face centered cubic **UiO-66** phase are preserved; peak attenuation/increased background after incubation in BHW is consistent with slight surface hydration rather than phase change. (d) SEM image of pristine **UiO-66** octahedra illustrating narrow size distribution and sharp facets. (e) ATR-FTIR spectra of pristine and 7-day BHW-aged **UiO-66** highlighting linker vibrations (ν(C=O) of carboxylate, aromatic C=C, ν(C–O)) and Zr–O(H) modes. **UiO-66** in BHW shows a subtle change near 1360–1400 cm^{-1} consistent with surface hydration/carbonate uptake.

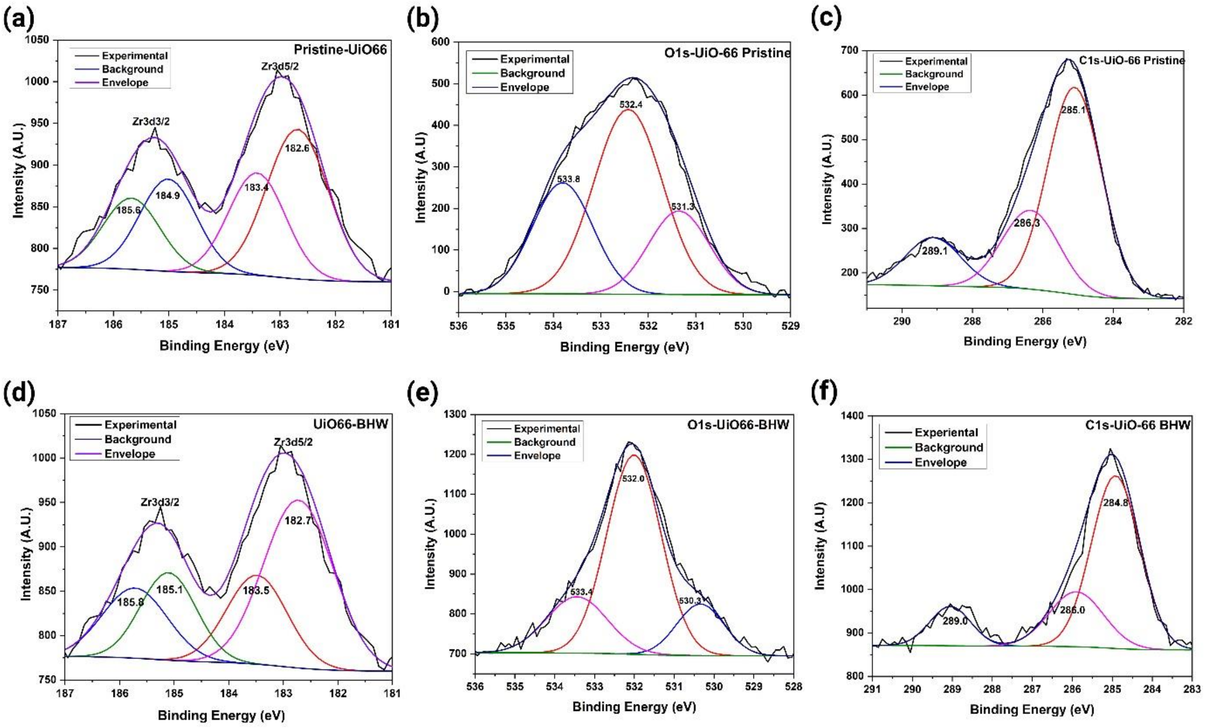

Figure S2. High-resolution XPS of UiO-66 before and after 7 days ageing in BHW. (a–c) Pristine **UiO-66**; (d–f) **UiO-66** after 7 d in BHW. (a,d) Zr 3d region fitted with the Zr^{4+} spin–orbit doublet ($3d_{5/2}$ ≈ 182–183 eV; $3d_{3/2}$ ≈ 184–185 eV). The doublet positions and area ratio remain consistent with Zr(IV); slight peak broadening after incubation in BHW indicates increased surface –OH/H_2O or adsorbates rather than Zr reduction. (b,e) O 1s deconvolution into contributions from μ_3-O/μ_3-OH of the Zr_6 node (~531–532 eV), carboxylate O of the benzenedicarboxylic acid (BDC) linker (~532–533 eV), and adsorbed H_2O/–OH (~533–534 eV). BHW ageing enhances the high-BE component, consistent with hydration/hydroxylation and/or carbonate uptake. (c,f) C 1s components assigned to C–C/C–H (~284.6 eV), C–O (~286 eV) and O–C=O (~288.6–289 eV). After BHW exposure the O–C=O contribution increases and a shoulder near ~289 eV becomes more evident, suggesting adventitious/bi-carbonate adsorption or surface carboxylate enrichment. Black lines: experimental spectra; green: background; coloured curves: fitted components; magenta: total fit (envelope).

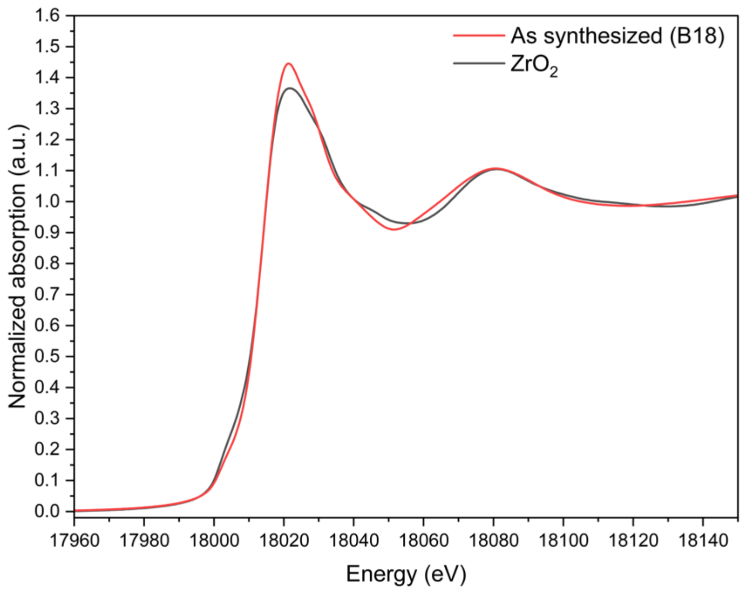

Figure S3. Comparison of Zr K-edge XANES Profiles. Zr K-edge XANES spectrum of as-synthesized **UiO-66** is compared with that of ZrO_2, a reference compound featuring Zr in the +4 oxidation state. B18 indicates the beamline used to perform QXAS experiment at Diamond Light Source, UK.

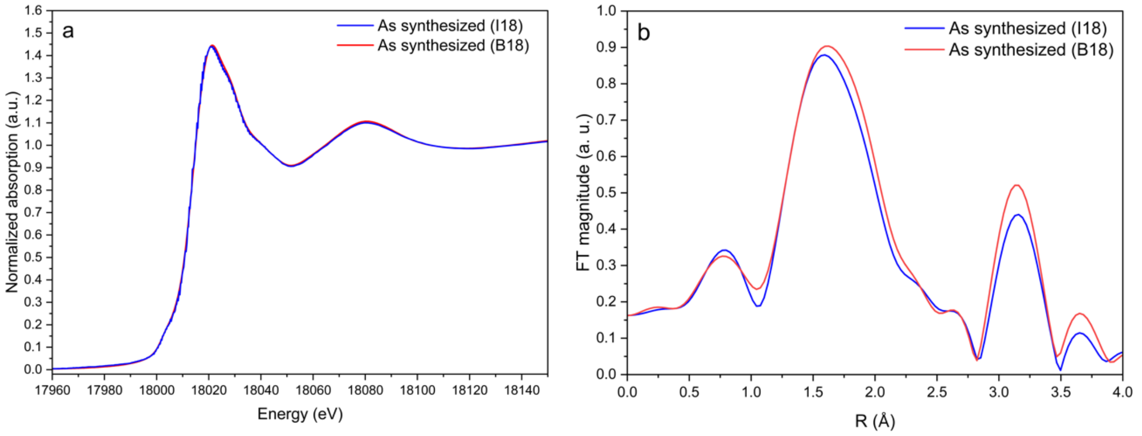

Figure S4. Comparison of Zr K-edge spectra. Zr K-edge **(a)** XANES and **(b)** Fourier-transformed EXAFS magnitudes for as-synthesized **UiO-66**, measured at the I18 and B18 beamlines of the Diamond Light Source. Data were collected at both beamlines to ensure reproducibility and to take advantage of their complementary capabilities: I18 provided microfocus mapping to localise Zr within biological samples, while B18 offered higher signal-to-noise bulk spectra for robust structural fitting. Including both ensures confidence in the interpretation of **UiO-66**'s local coordination environment.

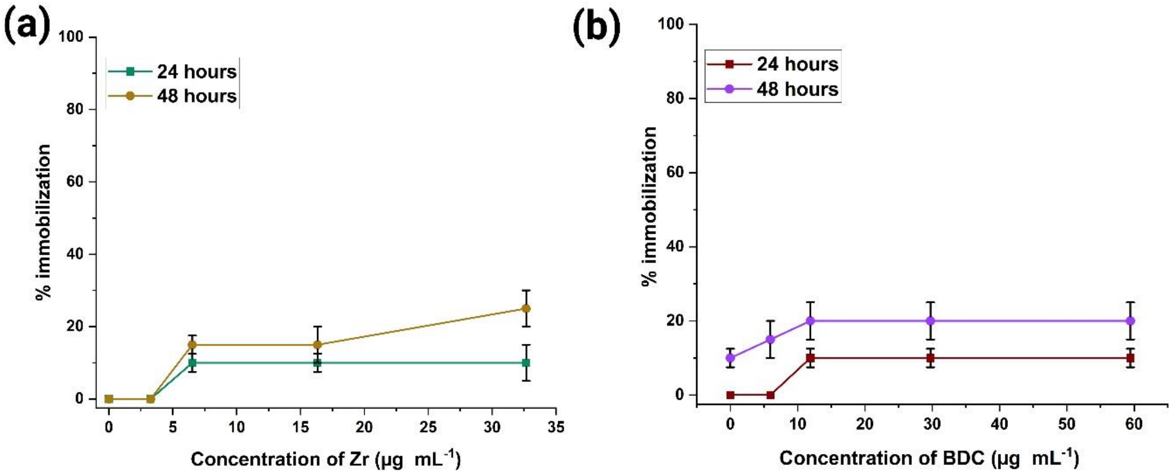

Figure S5. Acute immobilisation of *Daphnia magna* by UiO-66 constituents (OECD 202). (a) Percent immobilisation after 24 h (teal) and 48 h (gold) exposure to dissolved Zr (concentration expressed as µg mL^{-1} Zr). (b) Percent immobilisation after 24 h (red) and 48 h (purple) exposure to the terephthalate linker (BDC) (µg mL^{-1} BDC). Error bars represent SD based on 8 replicates (2 experiments each with 4 exposures per concentration). Immobilisation remained low across the ranges tested and increased only modestly at 48 h.

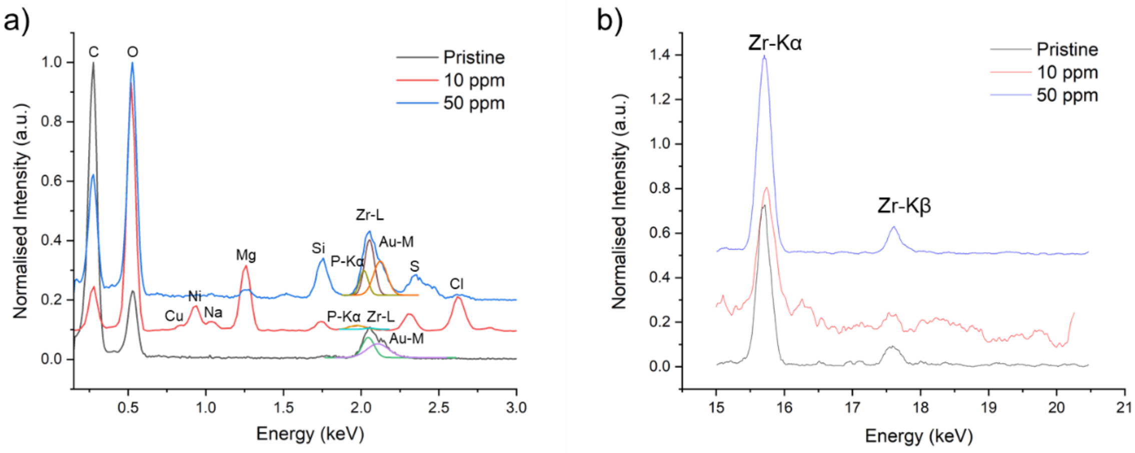

Figure S6. STEM-EDS spectra of pristine and depurated UiO-66. **(a)** Low-energy region (0–3 keV) showing C Kα (~0.28 keV), O Kα (~0.53 keV), and the emergence of P Kα (~2.01 keV) in depurated samples (10 and 50 μg mL^{-1}), absent in pristine material**. (b)** High-energy region (15–21 keV) highlighting Zr Kα (~15.8 keV) and Kβ (~17.7 keV); signal intensity increases with exposure concentration, reflecting Zr-rich deposits in depurates. Together with the elemental maps (Fig. 3), these spectra support phosphate-associated Zr phases forming during transit through the *D. magna* gut. (Note: EDS is semi-quantitative; thickness and matrix effects preclude absolute stoichiometry, but peak presence and co-variation are robust). Unit conversion- ppm= μg mL^{-1}

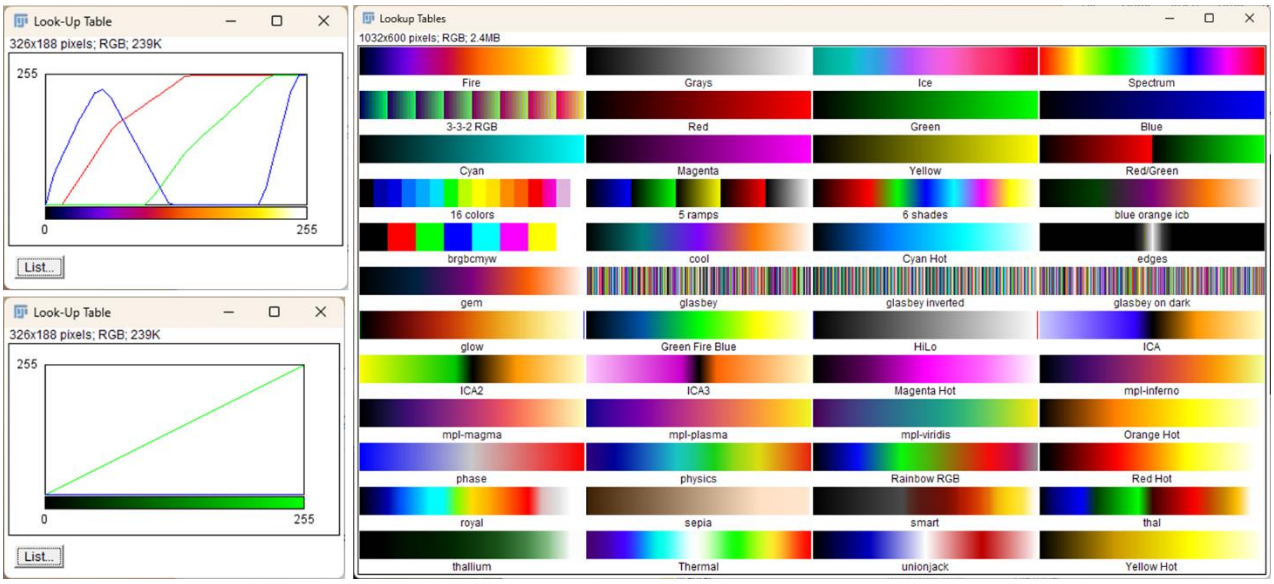

Figure S7: Micro-XRF maps of *Daphnia magna* showing zirconium and calcium distributions. Lyophilised daphnid after 24 h exposure to **UiO-66** (100 µg mL^{-1}) mapped at the Diamond Light Source microfocus beamline (~2.5 µm beam). **Zr** is rendered with the **Fire** LUT (warm colours = higher counts) and is strongly confined to the digestive tract (gut lumen/epithelium), consistent with ingestion and gut-level processing of the MOF. **Ca** is rendered with the **Green** LUT and delineates the calcified carapace and appendages, providing an anatomical reference. Colour scales are independently normalised to each channel's maximum. Elemental signals correspond to the **Zr-Kα** and **Ca-Kα** lines; higher intensities indicate greater areal concentrations.

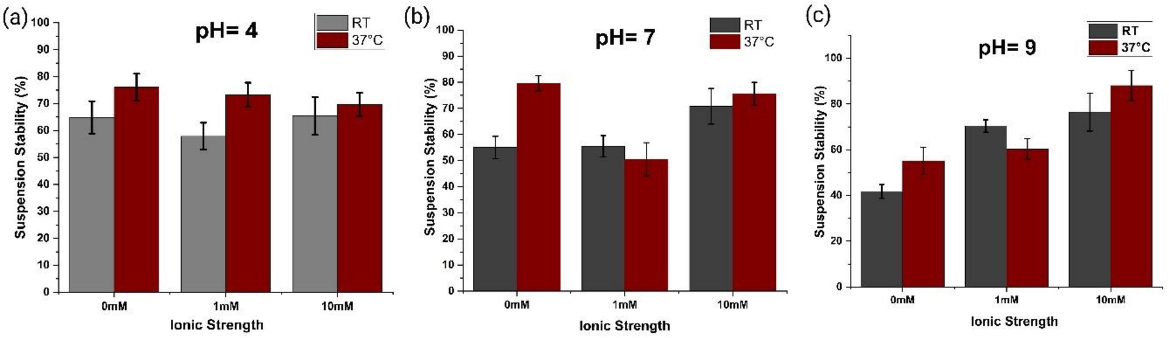

Figure S8. OECD TG 318 suspension stability of **UiO-66** as a function of ionic strength at pH 4 (a), pH 7 (b), and pH 9 (c), measured at room temperature (RT) and 37 °C. Stability is the % retained in the top layer at 6 h after the ∼1 μm centrifugation cut-off, normalized to the measured 0 h value; Zr was quantified by ICP-MS (NexION 350; He KED). Bars show mean ± SD (n = 3). Across pH, stability generally improves at 37 °C and at 10 mM $Ca(NO_3)_2$, with a minimum near 1 mM at pH 4–7, while the highest retention (~80–90%) occurs at pH 9, 10 mM, 37 °C.

Table S1. Composition, properties, and environmental/biological relevance of BHW used for **UiO-66** exposure experiments.

**Key Components / Composition**	**Typical pH**	**Environmental / Biological Relevance**	**Notes / Source**
Ca^{2+} (~60 mg/L), Mg^{2+} (~30 mg/L), Na^{+}, Cl^{-}, HCO_3^{-}, NO_3^{-} (<10 mg/L), trace metals (Fe, Mn), low DOC	~6.5–7.2	Natural moderately hard groundwater; relevant for environmental stability studies in freshwater aquifers and natural exposure scenarios	Collected from University of Birmingham borehole.<br>Low organic content but elevated hardness may stabilise / displace framework ligands
